# Bedside Removal of a Retained Plastibell Ring Using Handheld Electrocautery in the Emergency Department

**DOI:** 10.7759/cureus.92578

**Published:** 2025-09-17

**Authors:** Benjamin H Press, Elizabeth Chu, Emily S Blum

**Affiliations:** 1 Department of Pediatric Urology, Children's Healthcare of Atlanta, Atlanta, USA; 2 Department of Urology, Emory University, Atlanta, USA; 3 Department of Pediatric Urology, Georgia Urology, Atlanta, USA

**Keywords:** bedside procedure, circumcision complication, electrocautery, emergency department, pediatric circumcision, plastibell circumcision, retained ring, urologic foreign body

## Abstract

We report a 15-day-old infant with a proximally migrated Plastibell and marked glans edema. Ligature release with gentle manipulation was unsuccessful; scissor division was judged unsafe, and a ring cutter was unavailable. A handheld, battery-powered fine-tip cautery was used at the bedside to melt and divide the plastic ring with tissue protection and local analgesia, permitting removal without sedation. Postremoval examination showed preserved perfusion without urethral injury, and five-month follow-up demonstrated normal healing and cosmesis. This case suggests that handheld cautery may be a feasible and resource-conscious alternative when mechanical methods are unsuitable or unavailable. Careful patient selection and thermal safety measures are essential, and further experience is needed to define their role.

## Introduction

The Plastibell device is widely used for neonatal circumcision because it is simple to apply and generally well tolerated. The ring is designed to detach spontaneously within several days; contemporary series report typical separation around one week, which helps define when a device should be considered “retained” [1]. Retained or proximally migrated rings are a recognized complication that can lead to edema and urinary obstruction. In the event of delayed presentations, retained rings can cause penile incarceration or tissue injury, underscoring the need for timely and effective bedside management [2,3].

Multiple strategies for removal have been described, including release of the ligature with gentle manipulation, lubrication, or osmotic reduction to decrease edema, and mechanical division of the plastic ring using scissors or dedicated ring-cutting instruments; emergency department case reports provide step-by-step guidance for ring-cutter removal when simple measures fail [4,5]. Real-world cohort data also show that retained ring is among the most common Plastibell complications and that event rates are higher in infants than neonates, which explains the practical need for reliable bedside options [6].

We report a 15-day-old infant with a retained, proximally migrated Plastibell ring and marked edema in whom scissor division was deemed unsafe, and a ring cutter was unavailable. A handheld, battery-powered fine-tip cautery device was used to melt and divide the plastic ring at the bedside, permitting atraumatic removal without sedation. Based on our literature review, published bedside divisions of retained Plastibell rings predominantly use mechanical tools; we did not identify prior reports describing handheld cautery as the primary method of plastic ring division [4-6]. Because ring devices remain common, strategies for managing ring-specific complications remain clinically relevant [7].

## Case presentation

**Figure 1:**
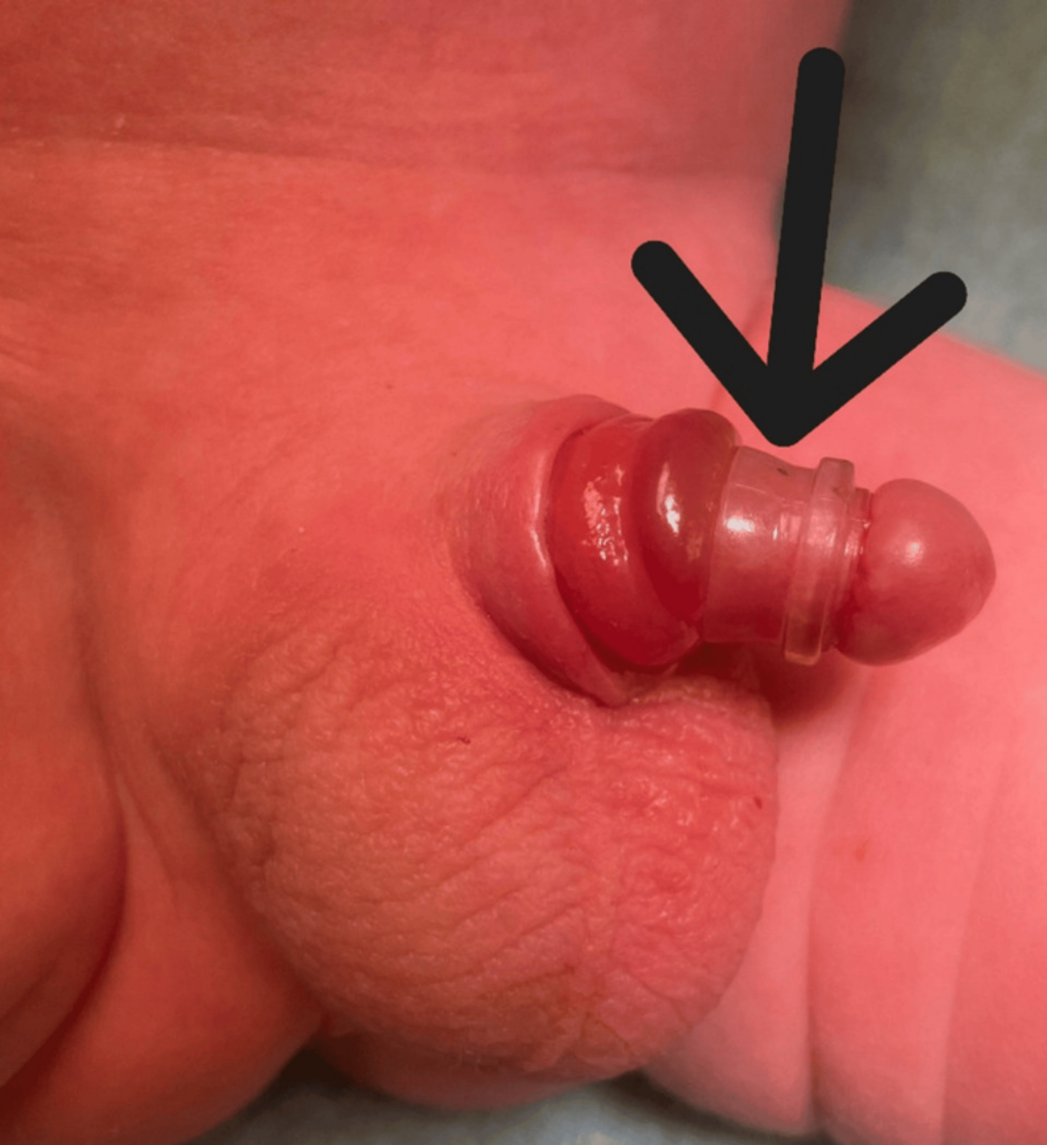
Initial examination demonstrating a malpositioned, retained Plastibell device (arrow) associated with marked glans edema Image courtesy: Used with permission from the patient's mother

A 15-day-old male infant, born at term and previously healthy, presented to the pediatric emergency department with a retained Plastibell circumcision device. The procedure had been performed on the second day of life. There was no bleeding or urinary retention. Before the urologic consultation, an attempt was made to manually remove the device by cutting the string to release the ligature, but the device did not separate. Initial examination demonstrated that the Plastibell device had migrated over the glans (Figure 1).

**Figure 2:**
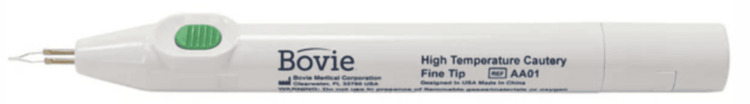
Portable electrocautery device used for removal of the Plastibell device Image courtesy: Reused with permission from the owner (https://mfimedical.com/products/bovie-disposable-high-temperature-fine-tip-cautery)

There was some discoloration to the corona of the penis that was tucked under the Plastibell ring. There was significant edema of the glans, most notably in the glans distal to the ring. Removal with scissors was deemed unsafe given the deep embedding and risk of iatrogenic injury. A ring cutter was considered but not immediately available, and its bulk would have posed a challenge given the significant edema and small operative field. The decision was made to attempt removal using a handheld battery-operated cautery device (Bovie Disposable High Temperature Fine Tip Cautery, MFI Medical, San Diego, CA) (Figure 2).

**Figure 3:**
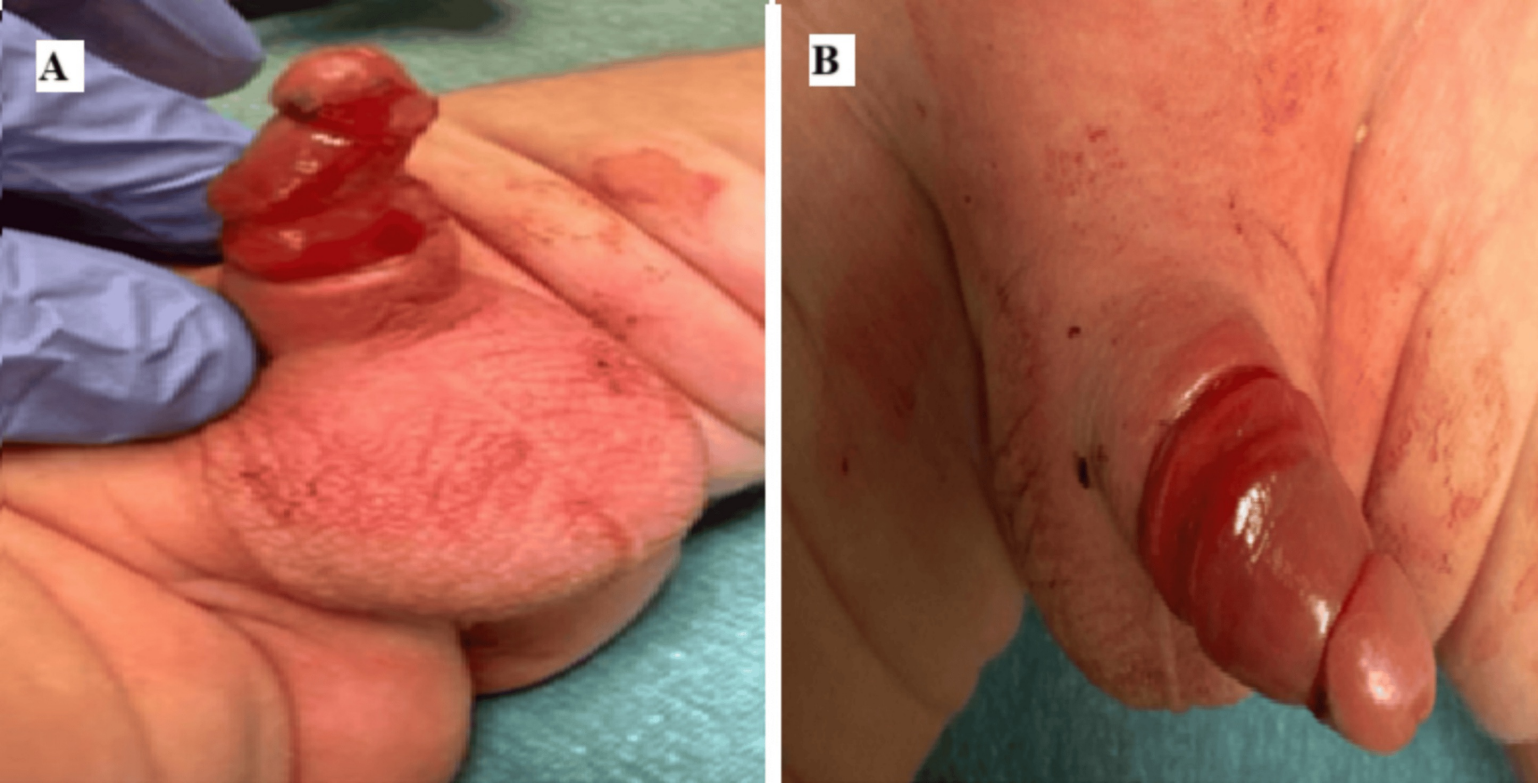
Examination immediately following removal of the Plastibell device. (A) Lateral view. (B) Dorsal view Significant distal glans edema is present following removal, but without vascular compromise, necrosis, or urethral injury Image courtesy: Used with permission from the patient's mother

With appropriate analgesia and tissue protection using a wet gauze barrier, the cautery device was used to carefully melt through the plastic ring. Cautery was used in brief (less than three seconds) intermittent activations with several seconds of cooling between bursts to limit heat conduction. After careful application of electrocautery for a total of five minutes, the device was successfully divided and removed in one piece. The patient tolerated the procedure well without sedation or complications. Postremoval examination revealed significant glans edema but no signs of vascular compromise, tissue necrosis, or urethral injury (Figure 3).

The area was cleaned and treated with Bacitracin ointment. The family received thorough discharge instructions and was advised to follow up with their primary care provider. The patient was discharged in stable condition. Five months following removal of the device, the glans has healed normally, and there are no parental concerns with cosmesis.

## Discussion

This report describes the successful bedside division of a retained Plastibell ring using a handheld fine-tip cautery device after conventional mechanical approaches were judged unsafe or unavailable. The existing literature predominantly details mechanical strategies, including ligature release through manipulation, scissor division, and the use of orthopedic or umbilical-cord ring cutters [4,5]. Within this context, our experience adds a resource-conscious option when edema, tissue entrapment, or equipment limitations preclude previously described methods. To our knowledge, this is the first reported case describing the use of handheld electrocautery for the removal of a retained Plastibell device. A review of the available literature revealed no prior similar cases.

Technique selection should be individualized. Each technique carries distinct benefits, risks, and resource requirements. Scissors are ubiquitous but may confer a higher risk of iatrogenic laceration when the ring is deeply embedded or visualization is compromised by circumferential edema. Ring cutters provide focused mechanical force and are well documented in stepwise emergency department guidance and small case series [4,5]; however, they are not uniformly stocked and can be cumbersome in a confined field. Operative removal maximizes visualization and tissue protection at the cost of anesthesia exposure and operating room resources. By contrast, handheld fine-tip cautery allows incremental thermoplastic weakening of the ring and precise division with minimal traction, potentially facilitating removal without sedation in selected presentations. Although we did not identify prior reports describing handheld cautery as the primary bedside method to divide a retained Plastibell ring, electrocautery is used in related contexts to cut or segment non-tissue foreign bodies (e.g., endobronchial electrocautery snares for impacted airway objects) when mechanical techniques are impractical, supporting the plausibility of a controlled, device-directed thermal approach [8,9].

Timely identification and triage of delayed ring separation are also important. Most Plastibell devices separate within approximately one week, and systems that prompt reassessment beyond this interval can expedite intervention and reduce complication risk [1,10]. Proximal migration with incarceration or evidence of tissue compromise (e.g., dusky glans, bleeding from the meatus, or obstructive symptoms) should prompt urgent evaluation and, when indicated, operative removal to mitigate the well-described risks of fistula formation and partial glans necrosis [2,3].

Thermal safety is paramount. Although the cautery was directed exclusively at the plastic ring in our case, any technique employing heat risks collateral thermal injury if energy is not meticulously confined. Recommended safeguards include interposing wet gauze or a thin metallic barrier between ring and tissue, using brief intermittent activations, avoiding continuous traction near the corona, and employing suction for smoke management. Contemporary reports of postcircumcision penile ischemia further justify a cautious approach and a low threshold for escalation when there is concern for vascular compromise [11].

Beyond technique selection, clear parent-facing education is essential; contemporary summaries of normal and abnormal post-Plastibell features provide practical visuals to reduce unnecessary returns while flagging true complications [12].

This report has limitations. It reflects a single case with an uncomplicated course; thus, external validity and generalizability are necessarily limited. Procedural success likely depends on operator familiarity with handheld cautery and adherence to thermal-protection measures. Comparative effectiveness vs. mechanical techniques cannot be inferred from this experience alone and warrants confirmation in additional cases or case series.

In summary, handheld fine-tip cautery may be considered as a selective bedside option for retained Plastibell rings when mechanical division is unsafe or unavailable, provided that rigorous thermal-safety precautions are observed and institutional protocols support its use. Further reports are needed to delineate indications, standardize procedural safeguards, and compare outcomes with established mechanical approaches [4-6].

## Conclusions

Retained Plastibell rings, though uncommon, can cause significant morbidity if not addressed promptly. This case shows that handheld fine-tip cautery may be a feasible bedside option for removal when mechanical methods are unsafe or unavailable. Use should be limited to carefully selected cases with strict tissue protection and operator familiarity, with a low threshold for operative escalation. Further reports are needed to define indications and compare outcomes with established mechanical approaches.
